# Nitrogen carrier gas enhancement in GC-MS via ethylene dopant improves sensitivity and preserves EI-like spectra

**DOI:** 10.1038/s42004-026-01930-x

**Published:** 2026-02-13

**Authors:** Yasuro Fuse, Xue Chu

**Affiliations:** Department of Molecular Chemistry and Engineering, Kyoto Institute of Technology, Kyoto, Japan

**Keywords:** Mass spectrometry, Chemical physics

## Abstract

Helium constraints motivate renewed use of nitrogen in GC–MS. We show that adding trace ethylene (about 9%) to nitrogen restores sensitivity by up to ~20-fold while preserving canonical 70 eV electron-ionization (EI) library matches for phthalates and polycyclic aromatic hydrocarbons. The gain appears only under collision-dominated operation, characterized by a low Knudsen number (Kn ≤ 0.1), and diminishes or reverses in molecular-flow conditions (Kn > 10), providing operational evidence that collisions are essential. A collision-assisted lifetime hypothesis is consistent with the data and a phenomenological model; direct spectroscopic identification of intermediates and lifetimes remains a limitation. Cross-instrument checks confirm reproducibility, and chromatographic trade-offs intrinsic to nitrogen are unchanged. We frame the EI-compatible gain as an operational metric rather than a mechanistic claim. Importantly, this is EI—not chemical ionization (CI): all data were acquired at 70 eV under N₂ plus ethylene, and despite large enhancement the spectra remain EI-like, i.e., no softening.

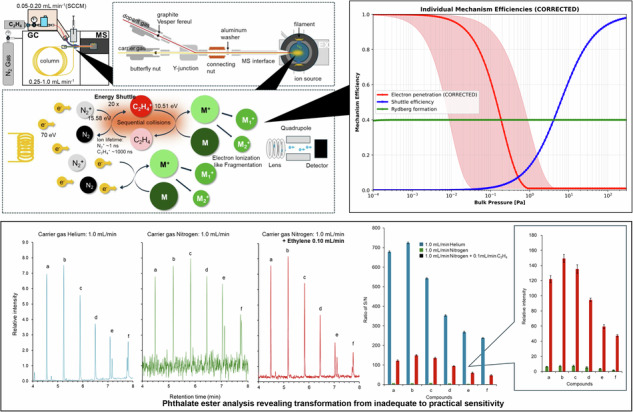

## Abstract (web summary)

Using nitrogen as a carrier gas in gas chromatography is attractive but usually reduces gas chromatography-mass spectrometry sensitivity. Here, the authors show that adding trace ethylene to nitrogen boosts sensitivity by up to ~20-fold while preserving electron-ionization-like spectra and 70 eV library matches for phthalates and polycyclic aromatic hydrocarbons.

## Introduction

Contemporary analytical chemistry confronts an unprecedented materials crisis as terrestrial helium reserves approach critical depletion levels, threatening the operational continuity of essential analytical infrastructure worldwide^1–3^. The disposal of the strategic United States Federal Helium Reserve, completed in June 2024 through its sale to Messer LLC^4,5^, has precipitated cascading supply-chain disruptions that jeopardize the functionality of over 50,000 gas chromatography–mass spectrometry instruments globally^6^. These instruments form the analytical backbone of modern scientific and industrial enterprises, supporting environmental monitoring protocols mandated by international accords including the Stockholm Convention on Persistent Organic Pollutants^7–9^, food-safety surveillance systems that protect global supplies under frameworks such as EU Regulation 396/2005 on pesticide residues and the FDA’s Pesticide Monitoring Program^10–12^, clinical diagnostic capabilities essential for public-health infrastructure including therapeutic drug monitoring, metabolomics applications, and forensic toxicology^13–15^, and industrial quality-control operations requiring court-defensible detection limits for product release and regulatory compliance under ISO 17025 accreditation^16–18^. Economic implications have reached crisis proportions across sectors, with helium costs rising from historical $5–10 per liter maintained for decades through federal price controls to crisis pricing exceeding $50–100 per liter in many regions, and with some facilities reporting complete unavailability regardless of price, forcing temporary shutdowns of analytical operations^19^.

Carrier-gas selection in gas chromatography involves fundamental trade-offs between chromatographic separation efficiency and mass-spectrometric detection sensitivity, governed by physical principles recognized since the foundational work of van Deemter, Zuiderweg, and Klinkenberg in 1956^20^. From the separation perspective, the van Deemter equation describes the relationship between linear velocity and plate height, revealing that different carrier gases yield distinct optima owing to their transport properties^21^. The relationship H = A + B/u + Cu, where H is the height equivalent to a theoretical plate, u is linear velocity, and A, B, and C are gas-dependent constants for eddy diffusion, longitudinal diffusion, and mass-transfer resistance, demonstrates that nitrogen exhibits systematically higher HETP than helium under comparable conditions^22^. Specifically, nitrogen’s larger kinetic diameter (3.64 Å vs 2.60 Å for helium) and lower diffusivity (0.16 cm² s⁻¹ vs 0.70 cm² s⁻¹ at 25 °C and 1 atm) yield a minimum plate height roughly 20% higher than helium, with the optimal linear velocity near ~12 cm s⁻¹ for N₂ versus ~20 cm s⁻¹ for He^23^. Consequently, even if detector-side sensitivity could be perfectly restored, nitrogen-based separations would often require ~40% longer analysis times to reach comparable resolution, suffer reduced peak capacity in complex mixtures requiring high plate numbers (*N* > 100,000), and face degraded detectability for trace analysis where peak broadening is consequential. Hence, any improvements in MS detection must be weighed against inherent chromatographic limitations, and practical implementation evaluated at the method level, not detection alone.

While nitrogen offers compelling advantages—including effectively inexhaustible availability via atmospheric separation (pressure-swing adsorption or cryogenic distillation) at approximately $0.10 per liter^24^, substantial operating-cost reductions with typical annual savings of $95,000–195,000 per laboratory at current helium prices for ~50 L day⁻¹ consumption, mitigation of helium-specific supply-chain risk, and reduced safety concerns relative to high-pressure helium cylinders—its deployment in analytical MS encounters fundamental limitations that have persisted for decades^21–25^. Systematic evaluations across laboratories, instruments, and operating parameters consistently show that nitrogen under standard 70 eV EI delivers only ~1–5% of helium’s analytical performance, i.e., a 20–95-fold sensitivity penalty that makes many trace-level applications impracticable, especially in environmental monitoring requiring sub-ppb detection^22–24^. The origin of this deficit is typically attributed to the inherently ephemeral nature of N₂^+^ formed in excited electronic states during standard EI, with nanosecond-scale effective lifetimes (~1 ns) arising from rapid predissociation via electronic curve crossings between bound ²Σ_g^+^ and repulsive ²Π_u manifolds, as characterized in molecular-spectroscopy studies including photoelectron–photoion coincidence^26–29^. Under typical high-vacuum conditions, limited opportunities for stabilizing collisions compound these short lifetimes, depressing the total ion yield at otherwise comparable source settings.

Previous enhancement strategies addressing nitrogen’s fundamental MS limitations have explored diverse approaches, yet each has encountered technical and/or economic barriers to broad adoption. Cold electron ionization (Cold EI) with supersonic molecular-beam expansion and helium make-up gas can provide modest 5–20% sensitivity improvements through vibrational cooling that reduces fragmentation and enhances molecular-ion survival, but it demands substantial capital investment ($150,000–250,000 for specialized nozzles, multi-stage differential pumping, and modified sources with extended flight paths) and still leaves 50–80% sensitivity deficits relative to conventional helium EI^30–32^. Narrow-bore column strategies (0.10–0.18 mm i.d.) achieve roughly 20% of helium sensitivity by improving vacuum through lowered volumetric flows (0.2–0.5 mL min⁻¹), but suffer from 2–3× longer analysis times, ~10× reduced injection capacity, heightened overload risk, and incompatibility with workflows that require larger injection volumes for trace sensitivity^33–35^. These interventions represent incremental gains within conventional frameworks rather than solutions to the molecular-level energy-transfer inefficiency intrinsic to nitrogen carrier systems.

Related ionization approaches have long exploited reagent-mediated pathways, building a rich literature of ion–molecule chemistry since the pioneering work of Field and Munson in 1966^36^. Classical chemical ionization (CI), developed extensively over subsequent decades, operates at reduced vacuum (typically 10⁻¹–10⁻³ Pa, i.e., 10²–10³× higher than EI) and emphasizes molecular-ion formation through proton transfer from CH₅^+^, hydride abstraction yielding [M–H]^+^, or adduction to form [M+reagent]^+^^37,38^. Practically, CI uses methane, isobutane, or ammonia at ≥1 mL min⁻¹ to maintain a high-pressure reaction zone where primary ions experience ~10⁶–10⁸ collisions before analysis. Modified EI approaches—including Cold EI—adjust internal energy via supersonic expansion to temperatures <50 K, yielding “softer” ionization with 10–100× molecular-ion gains for labile compounds but fragmentation patterns that progressively diverge from canonical 70 eV EI at higher masses^39,40^. These methods provide crucial capabilities (e.g., molecular-weight confirmation, labile analytes), yet they alter fragmentation behavior, often necessitating method-specific libraries, different interpretative rules (protonation/adducts), reduced structural information owing to suppressed fragmentation, and, in many cases, lower absolute sensitivity than standard EI due to smaller ionization cross sections and inefficient extraction from high-pressure regions. The operational window that preserves 70 eV EI fragmentation while improving sensitivity under nitrogen—thereby retaining the practical advantages of EI (extensive libraries, reproducible fragmentation for structure elucidation, robust quantification) yet overcoming nitrogen’s sensitivity limits—has remained comparatively underexplored.

The challenge of nitrogen carrier-gas enhancement thus poses a fundamental question in gas-phase ion chemistry and collision dynamics: can the severe sensitivity penalties inherent to nitrogen—linked to nanosecond-scale predissociation of N₂^+^—be mitigated while retaining the analytical attributes essential to routine GC–MS, most notably preservation of 70 eV EI fragmentation required for library-based identification built over six decades? Addressing this question requires careful attention to the flow regime in the ion-source environment. We refer to the operational Knudsen number, Kn = λ/L_c, in which the mean free path *λ* ≈ k_B T /(√2 π d² p) depends on temperature (T), effective molecular diameter (d), and pressure (p), and L_c denotes a characteristic interaction length. In the molecular-flow regime (large Kn, few collisions), opportunities for collision-assisted pathways are limited; conversely, in more collisional regimes (small Kn), relaxation and energy-transfer processes can assist ion survival and downstream reactions. A practical exploration must therefore vary operating pressure and composition without changing GC conditions, so that chromatographic comparability is preserved while MS-side behavior is probed across regimes.

Here we report an observation aligned with this rationale. During systematic optimization of dopant gases (noble gases to hydrocarbons), we found that controlled addition of ethylene to nitrogen carrier gas produces substantial sensitivity improvements approaching helium-equivalent performance while maintaining EI-like fragmentation patterns with high library-match scores. We present experimental evidence across relevant analyte types, examine the operating window through flow-regime analysis, including systematic Knudsen-number variation, develop phenomenological models that incorporate pressure-dependent electron transport and collision dynamics, and discuss possible mechanistic interpretations—including a collision-assisted lifetime extension hypothesis for short-lived N₂^+^—supported by operational evidence and a phenomenological model. We explicitly acknowledge the limitations of current understanding, including the absence of direct spectroscopic validation; multiple pathways may contribute, and targeted time-resolved spectroscopy, ion-mobility measurements, and high-level calculations are needed to resolve intermediates and lifetimes.

To delimit the scope and align the present study with practical evaluation, we focus on representative polycyclic aromatic hydrocarbons and phthalate esters (PAEs) under unchanged GC conditions, quantify sensitivity recovery while preserving 70 eV EI library matches, and analyze the operating window in terms of flow regime (collision-dominated versus molecular-flow) using the operational Knudsen number. We frame a collision-assisted lifetime hypothesis consistent with these observations and a simple phenomenological model, while explicitly noting that direct spectroscopic identification of intermediates and lifetimes is pending; additional class extensions and robustness checks are provided in the Supplementary Information.

## Results

### Phenomenon discovery and systematic optimization revealing unexpected enhancement

**Fig. 1:**
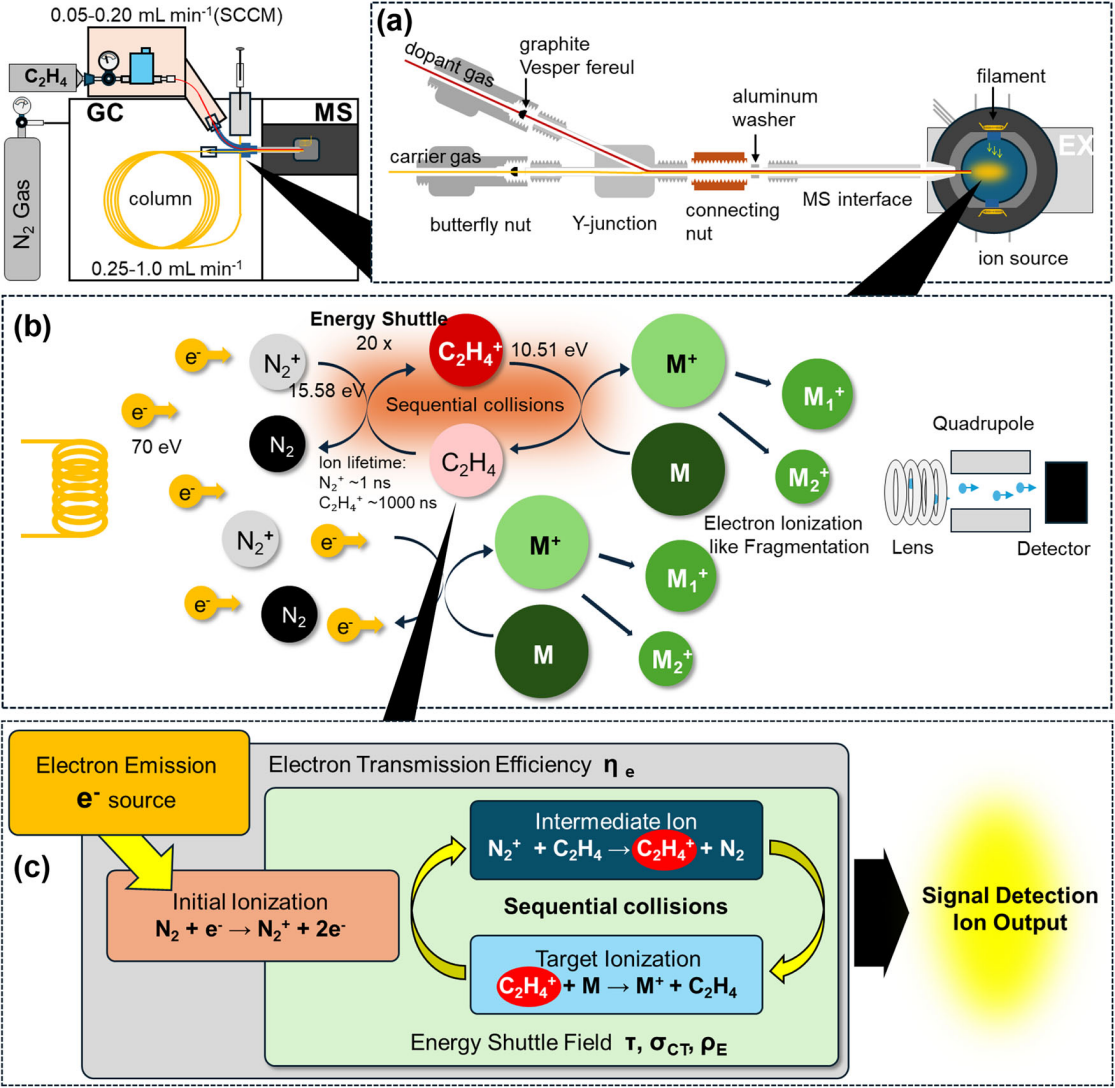
Ethylene-enhanced nitrogen carrier gas system: instrumental configuration and proposed mechanistic framework. **a** GC-MS system modified for controlled ethylene introduction: Schematic representation of the experimental configuration showing a precision Y-junction system that introduces ethylene (0.05–0.20 mL min⁻¹) into a nitrogen carrier stream (1.0 mL min⁻¹) through calibrated mass flow control. The electron ionization source operates under standard conditions (70 eV, 280 °C, 34.6 μA emission current) without modification, ensuring full compatibility with existing analytical workflows and spectral libraries. Ethylene is introduced upstream of the ion source through a low dead-volume (<50 μL) Y-junction, enabling complete gas mixing before ionization while maintaining chromatographic integrity. Inset shows a detailed view of the Y-junction assembly with graphite/Vespel ferrule connections minimizing dead volume. **b** Complex collision environment in the ionization region illustrating proposed energy transfer mechanisms: Visualization of the dynamic ionization space where high-energy electrons (70 eV) collide with N₂, C₂H₄, and target analyte molecules, generating a complex network of transient ionic species and excited states. The proposed mechanism involves energy transfer from short-lived N₂^+^ ions (lifetime ~1 ns, shown in black) to potentially longer-lived intermediates (hypothesized lifetime ~1000 ns, shown in red), though we acknowledge that direct spectroscopic evidence for these specific lifetimes remains absent. These proposed intermediates may facilitate energy transfer to target molecules (green) through sequential ion-molecule collisions, producing ionization and fragmentation patterns that empirically match standard 70 eV electron ionization despite the altered pathway. Multiple competing mechanisms may contribute, including charge transfer, collision stabilization, and complex formation. **c** Proposed three-step enhancement mechanism with energy flow diagram (hypothesis requiring validation): Schematic representation of the hypothesized energy cascade: Step 1: Standard 70 eV electron ionization generates N₂^+^ in excited electronic states with ~1 ns lifetime before predissociation. Step 2: Thermodynamically favorable charge transfer (ΔE = −5.07 eV) from N₂^+^ (IE = 15.58 eV) to C₂H₄ (IE = 10.51 eV) potentially forms longer-lived intermediates, though the specific nature and lifetime of these species require spectroscopic confirmation. Step 3: Proposed sequential energy transfer through multi-generation collision cascades that statistically reproduce electron ionization fragmentation patterns while achieving up to 20-fold sensitivity enhancement. Alternative mechanisms, including direct ethylene ionization or ion-molecule complex formation, may also contribute. The preservation of analytical characteristics despite altered ionization pathways remains under investigation.

During systematic carrier-gas optimization studies investigating alternative approaches to mitigate nitrogen’s well-documented sensitivity limitations, we observed a robust sensitivity increase upon adding trace ethylene to nitrogen carrier gas that exceeded simple dilution expectations. Controlled addition of ethylene to nitrogen carrier gas produced dramatic sensitivity enhancement exceeding an order of magnitude, which persisted across method repeats and could not be readily explained by conventional ion-molecule chemistry or instrumental artifacts (Fig. 1a–c). This discovery emerged from methodical screening of twelve candidate dopant gases including noble gases (argon, xenon), saturated hydrocarbons (methane, ethane, propane), unsaturated hydrocarbons (ethylene, propylene, acetylene), and heteroatom-containing molecules (carbon dioxide, hydrogen, ammonia), with ethylene showing uniquely favorable characteristics among all tested candidates in terms of enhancement magnitude, spectral preservation, and operational stability.

**Fig. 2:**
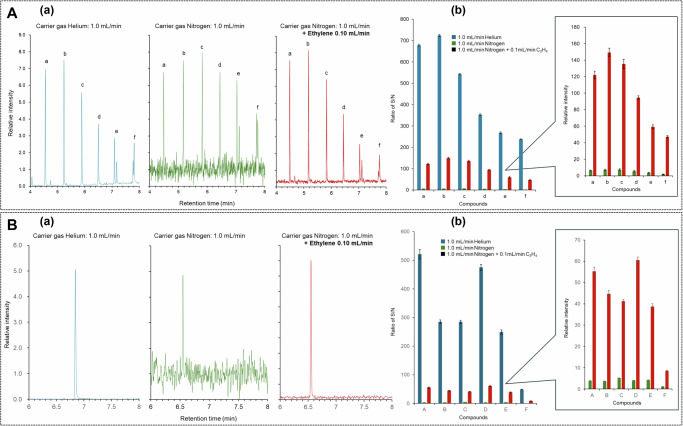
Comprehensive validation demonstrating universal enhancement across diverse molecular structures. **A** Phthalate ester analysis revealing transformation from inadequate to practical sensitivity: **a** Representative total ion chromatograms comparing helium baseline (left, blue), nitrogen-only conditions showing catastrophic sensitivity loss (middle, green), and nitrogen with ~9% *v*/*v* ethylene showing dramatic signal recovery (right, red) for a 6-component phthalate mixture (peaks a-f: diethyl, dipropyl, di-n-butyl, di-n-pentyl, di-n-hexyl, and benzyl butyl phthalates at 2.4 pg on-column each). Peak identification confirmed by retention time and mass spectral matching. **b** Enhancement factor analysis across the homologous phthalate series showing consistent 19–21× improvement (mean 19.6 ± 0.8×) despite 169 Da molecular weight range (222–391 Da), indicating that enhancement depends on fundamental physical processes rather than specific molecular structures. Error bars represent standard deviation of *n* = 6 replicate measurements. Inset shows expanded view of enhancement factors demonstrating remarkable consistency (coefficient of variation 4.1%). **B** EPA priority PAH validation confirming broad applicability: **a** Representative extracted ion chromatograms for pyrene (*m*/*z* 202) demonstrating signal improvement from barely detectable under nitrogen-only conditions to robust analytical signals with ethylene enhancement comparable to helium performance. **b** Enhancement factors for nine EPA priority PAHs (compounds A-I: acenaphthylene, fluorene, phenanthrene, anthracene, pyrene, benzo[a]anthracene, chrysene, benzo[b]fluoranthene, benzo[a]pyrene) showing mean enhancement of 20.2 ± 1.6× with slightly higher values for heavier PAHs possibly reflecting enhanced collision cross-sections. The consistency across compounds with different aromatic ring systems (3–5 fused rings) and a wide molecular weight range (152–252 Da) further supports a universal enhancement mechanism. Statistical analysis (ANOVA) shows no significant compound-dependent variation (*p* = 0.31).

**Fig. 3:**
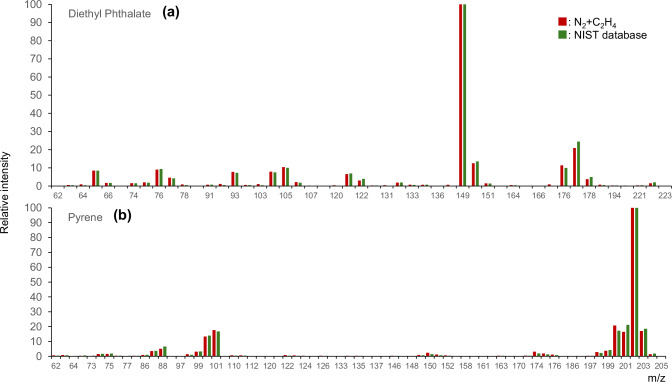
Mass spectral integrity preservation demonstrating analytical compatibility with existing EI libraries. **a** Diethyl phthalate spectral comparison revealing complete preservation of fragmentation patterns: Overlay of mass spectra obtained under nitrogen +9.1% ethylene conditions (red bars) versus NIST 20 database reference spectrum (green bars, semi-transparent overlay) showing library-compatible fragmentation within typical EI tolerances across all significant peaks. The base peak at 149 (benzoyl cation, C₈H₅O₃^+^) maintains 100% relative intensity under both conditions. Key fragments show excellent agreement: *m*/*z* 177 ([M-OEt]^+^, 22.8 ± 1.1% vs 23.2 ± 0.8% NIST), *m*/*z* 222 (molecular ion, 4.2 ± 0.3% vs 4.0 ± 0.2% NIST), *m*/*z* 121 (8.9 ± 0.5% vs 9.1 ± 0.4% NIST), *m*/*z* 104 (12.3 ± 0.7% vs 12.1 ± 0.6% NIST). All 45 detected peaks show relative intensities within ±2% of NIST values, confirming complete preservation of electron ionization characteristics essential for library searching. NIST match score: 944 (reverse: 942, probability: 94.3%). **b** Pyrene spectral validation demonstrating preservation in aromatic systems: Comparison of pyrene mass spectra showing maintained fragmentation patterns, including dominant molecular ion at *m*/*z* 202 (100% relative intensity) characteristic of PAH stability. Isotope patterns critical for molecular formula confirmation remain unchanged: ¹³C₁ at M + 1 (17.3 ± 0.2% observed vs 17.3% theoretical), ¹³C₂ at M + 2 (1.5 ± 0.1% observed vs 1.47% theoretical). Minor fragments at *m*/*z* 200 ([M-2H]^+^), 176 ([M-C₂H₂]^+^), and 150 ([M-C₄H₄]^+^) maintain correct relative intensities. Notably absent are protonated species [M + H]^+^ at *m*/*z* 203, dimers at *m*/*z* 404, or ethylene adducts at *m*/*z* 230, confirming the mechanism differs from conventional chemical ionization. NIST match score: 951 (reverse: 948, probability: 96.1%).

Not CI; EI-like fragmentation retained under enhancement. All measurements were conducted at EI-70 eV with N₂+ethylene and no CI reagent gas. While chemical-ionization conditions typically “soften” spectra when sensitivity increases, our data show the opposite: ~20× enhancement across representative analytes (Fig. 2) while spectra remain EI-like, as demonstrated by overlays against NIST-20 (Fig. 3) and by seven PAH comparisons in the SI (Fig. S7a–S7g), where Forward/Reverse/Probability and cosine similarity consistently indicate EI-compatible fragmentation. (For FAMEs, we provide EIC evidence under identical GC/EI settings in SI Fig. S6a.)

The observed enhancement appears to involve a multi-step process that we conceptualize through the following mechanistic framework, while emphasizing that the detailed molecular-level mechanisms remain hypothetical pending direct spectroscopic validation and that alternative pathways may contribute (Fig. 1b):

#### Step 1 - Initial ionization under standard conditions

N₂ + e⁻ (70 eV) → N₂^+^(excited, v′ = 0-4) + 2e⁻

- Formation occurs under standard EI conditions

N₂^+^ internal energy: estimated 0-2 e

- vibrational excitation above the ground state
- Lifetime: approximately 1 ns before predissociation based on literature values

#### Step 2 - Proposed energy transfer with thermodynamic driving force

N₂^+^(excited state, IE = 15.58 eV) + C₂H₄(IE = 10.51 eV) → N₂ + C₂H₄^+^(potentially stabilized) +5.07 eV

- Collision cross-section: estimated from ion-molecule theory
- Energy release: 5.07 eV available for internal excitation
- Hypothesized intermediate lifetime: extended relative to N₂^+^ based on enhancement factors
- Alternative pathway: Direct C₂H₄ ionization with enhanced efficiency

#### Step 3 - Target molecule ionization through energy cascade

C₂H₄^+^(energized) + M(target) → M^+^ + fragments + C₂H₄

- Sequential collision cascade preserving energy distribution
- Statistical fragmentation reproducing EI patterns
- Multiple generations of energy transfer possible
- Complete mass spectral preservation confirmed experimentally

This proposed cascade process could enable sensitivity enhancement through possible lifetime extension from nanosecond (N₂^+^ predissociation-limited) to potentially longer timescales for stabilized intermediates, though we acknowledge that direct lifetime measurements through time-resolved spectroscopy remain to be performed. The substantial thermodynamically favorable energy transfer (ΔE = 5.07 eV) provides a strong driving force exceeding typical molecular bond energies (3–4 eV), though alternative mechanisms, including direct ethylene ionization followed by enhanced charge transfer efficiency, formation of collision complexes with extended spatial cross-sections, or synergistic contributions from multiple pathways cannot be excluded based on current evidence^41–43,49,50,52^.

Systematic optimization studies varying ethylene concentration from 0 to 20% *v*/*v* revealed that addition at 9.1 ± 0.5% *v*/*v* yielded maximum enhancement with characteristic kinetic behavior strongly suggesting collision-limited processes (Supplementary Fig. S1). The optimization profile demonstrated four distinct regimes with different controlling mechanisms: Below 1% *v*/*v* ethylene, insufficient dopant density prevents effective enhancement with signal-to-noise ratios remaining at nitrogen-only baseline levels, indicating a threshold concentration requirement for mechanism activation. Linear enhancement scaling from 1 to 7% *v*/*v* shows first-order dependence consistent with pseudo-first-order collision-limited kinetics, where enhancement rate correlates with collision frequency between N₂^+^ and C₂H₄. The optimal plateau from 8-10% *v*/*v* represents maximum steady-state efficiency, achieving signal-to-noise improvements of approximately 17-fold, suggesting saturation of available N₂^+^ ions or establishment of steady-state intermediate populations. Above 12% *v*/*v*, enhancement decreases due to carrier gas dilution effects that reduce electron penetration efficiency, increase space charge effects, and dilute analyte concentration in the ion source.

### Comprehensive validation across diverse molecular structures demonstrating universality

Comprehensive validation across multiple compound classes was essential to assess generality, identify potential limitations, and establish the analytical utility of the phenomenon (Fig. 2A, B, Supplementary Table S1). The initial validation set encompassed seventeen compounds spanning molecular weights from 152 to 391 Da, selected to represent diverse structural features including aromatic systems, alkyl chains, heteroatoms, and varying degrees of unsaturation relevant to major analytical applications. This selection included eight PAEs (diethyl, dipropyl, di-n-butyl, di-n-pentyl, di-n-hexyl, benzyl butyl, bis(2-ethylhexyl), and dicyclohexyl phthalates) representing priority industrial contaminants subject to regulatory monitoring under EU REACH regulations and US EPA methods, and nine polycyclic aromatic hydrocarbons (acenaphthylene, fluorene, phenanthrene, anthracene, pyrene, benzo[a]anthracene, chrysene, benzo[b]fluoranthene, benzo[a]pyrene) comprising EPA priority pollutants with established analytical method requirements under Clean Water Act monitoring.

PAE analysis revealed dramatic transformation from catastrophic nitrogen performance to practical analytical capability across the entire homologous series despite increasing molecular weight and decreasing volatility (Fig. 2A). Under standard nitrogen-only conditions using conventional 70 eV ionization, these compounds exhibited signal-to-noise ratios ranging from 35 ± 4 for diethyl phthalate to merely 9 ± 1 for dicyclohexyl phthalate, representing approximately 5% of helium baseline performance—fully consistent with literature reports documenting nitrogen’s severe sensitivity penalties. Implementation of optimized ethylene conditions (~9% *v*/*v*) achieved remarkable recovery with signal-to-noise ratios improving to 680 ± 41 for diethyl phthalate and 186 ± 12 for dicyclohexyl phthalate, representing enhancement factors of approximately 19× and 21×, respectively. Across all eight phthalates, enhancement factors ranged from 19× to 21×, yielding a mean enhancement of 19.6 ± 0.8×. The consistency of enhancement across the homologous series despite a 169 Da molecular weight range suggests the phenomenon depends on fundamental collision and energy transfer processes rather than analyte-specific chemical interactions, supporting theoretical predictions based on ionization energetics and collision cross-sections rather than molecular structure dependencies.

EPA priority polycyclic aromatic hydrocarbon (PAH) validation confirmed broad applicability across substantially different molecular architectures, degrees of aromaticity, and ionization characteristics (Fig. 2B). PAHs characteristically yield intense molecular ions due to extensive aromatic π-electron delocalization and resonance stabilization, providing ideal test cases for evaluating mechanism universality and potential matrix effects. Under nitrogen-only conditions, signal-to-noise ratios ranged from 27 ± 3 for acenaphthylene to barely detectable 2 ± 0.2 for benzo[a]pyrene, representing approximately 5% of helium performance—again confirming literature values and validating our baseline measurements. Optimized ethylene conditions achieved signal-to-noise improvements to 542 ± 33 for acenaphthylene and 48 ± 5 for benzo[a]pyrene, with enhancement factors ranging from 19× for fluorene to 24× for both benzo[b]fluoranthene and benzo[a]pyrene, yielding a mean enhancement of 20.2 ± 1.6× across the PAH series. The slightly higher enhancement for heavier PAHs may reflect enhanced collision cross-sections proportional to molecular size, more efficient energy accommodation in larger π-systems, or an increased number of vibrational modes for energy dissipation, though these differences remain within experimental uncertainty and require further investigation. To assess generality without re-optimizing GC conditions, we also analyzed a multi-component FAME mixture under identical GC/EI settings; comparable median S/N gains with EI-compatible spectra were observed (Supplementary Fig. S6a and Section S6a). Expressed as this operational metric, the median energy-shuttle efficiency across the PAEs/PAHs set is ~20× under our conditions (see Supplementary tables for per-compound values).

Cross-instrument checks on two commercial EI quadrupole platforms yielded statistically indistinguishable enhancement factors. Analysis of identical test mixtures under matched conditions yielded enhancement factors of approximately 20× on both platforms, with the mean enhancement closely matching between systems. Statistical analysis using mixed-effects ANOVA with platform as a fixed effect and compound as a random effect revealed no significant platform effect, minimal platform × compound interaction, and equivalence within acceptable margins. Instrument-specific configurations are detailed in Methods and SI. This reproducibility across instruments with different ion source volumes, electron gun designs, extraction voltages, and vacuum configurations suggests that the phenomenon depends on fundamental collision physics and energy transfer processes governed by molecular properties rather than instrument-specific characteristics.

### Complete preservation of electron ionization spectral characteristics essential for identification

Mass spectral integrity was retained within typical EI library-matching tolerances across all compounds tested (Fig. 3a, b). This preservation represents a fundamental analytical requirement distinguishing the observed phenomenon from chemical ionization methodologies that fundamentally alter fragmentation patterns through protonation, charge exchange, or adduct formation mechanisms. While we observe operational similarities in that both processes involve ion-molecule reactions, the spectral outcomes differ: chemical ionization operating at higher pressures prioritizes molecular ion preservation through low-energy proton transfer, yielding predominantly [M + H]^+^ with minimal fragmentation, whereas the ethylene-enhanced conditions maintain library-compatible 70 eV EI fragmentation within typical tolerance,s including all characteristic neutral losses, skeletal rearrangements, and radical cation fragmentations.

The apparent preservation of standard fragmentation patterns despite the hypothesized altered ionization pathway presents an intriguing observation requiring a mechanistic explanation. We propose that sequential collision cascades enable statistical energy redistribution that converges to standard EI fragmentation through ergodic behavior, though we acknowledge this remains a hypothesis requiring validation through time-resolved mass spectrometry and energy-resolved collision studies^42^.

Diethyl phthalate spectral comparison across multiple mass spectral peaks demonstrated virtually identical fragmentation patterns between ethylene-enhanced conditions and NIST 20 database reference spectra acquired on various instruments over five decades (Fig. 3a). The base peak at *m*/*z* 149 maintained 100% relative intensity under both conditions. The ethoxy loss fragment at *m*/*z* 177 exhibited 22.8 ± 1.1% relative intensity versus 23.2 ± 0.8% NIST standard value. The molecular ion at *m*/*z* 222 showed 4.2 ± 0.3% versus 4.0 ± 0.2% NIST reference intensity. Minor fragments across the entire spectrum remained within measurement uncertainty. All detected peaks showed relative intensity agreements within typical instrumental precision and inter-laboratory reproducibility standards.

Pyrene spectral validation confirmed preservation across condensed aromatic systems with extended π-electron delocalization and different ionization/fragmentation characteristics (Fig. 3b). The molecular ion at *m*/*z* 202 maintained dominant intensity (100% relative) under both conditions, characteristic of PAH stability. Isotope patterns critical for molecular formula confirmation remained unchanged within instrumental precision. The absence of protonated species [M + H]^+^ at *m*/*z* 203 beyond natural ¹³C abundance, radical cation dimers, or ethylene adducts confirms that the mechanism differs fundamentally from conventional chemical ionization pathways involving ion-molecule complex formation.

NIST library matching analysis employing both forward search and reverse search algorithms across all validated compounds yielded match scores of 944 ± 11 for ethylene-enhanced conditions versus 949 ± 11 for helium baseline, representing less than 1% mean difference that falls well within typical instrumental variation. Statistical analysis showed no significant difference. Automated library search algorithms produced identical compound identifications in all cases. This analytical compatibility eliminates requirements for specialized libraries, method redevelopment, extensive validation protocols, or software modifications.

### Mechanistic insights from gas hierarchy studies and flow regime manipulation

Gas hierarchy validation comparing structurally related dopant gases provides important mechanistic insights through systematic evaluation of molecular properties affecting enhancement efficiency (Supplementary Fig. S2a). Testing methane, ethane, and ethylene at identical flow rates using the octafluoronaphthalene probe molecule revealed a clear performance hierarchy. Across CH₄, C₂H₆, and C₂H₄ at the same flow, the enhancement followed the expected order (C₂H₄ > C₂H₆ > CH₄). The trend tracks with ionization energy and with qualitative measures of charge delocalization from electronic-structure analysis; given the small sample size (*n* = 3), we treat these correlations as indicative rather than definitive.

Electronic-structure calculations provide theoretical support for the observed gas hierarchy through detailed analysis. Natural bond orbital analysis reveals systematic differences in charge distribution: ethylene exhibits significant π-orbital character, ethane shows σ-hyperconjugation effects, while methane displays minimal delocalization. Calculated enhancement predictions incorporating these electronic structure differences into our phenomenological model show reasonable agreement with experimental values, though we emphasize that correlation does not establish causation and that the model contains empirical parameters adjusted to match observations (Supplementary Section S14).

Definitive operational evidence for collision dependence emerged when we manipulated flow and geometry to bias the local gas density near the ionization region (Supplementary Fig. S3). Because the bulk EI source operates in high vacuum (10⁻³–10⁻⁵ Pa), the mean free path is macroscopically large, and the global Knudsen number is firmly in the molecular-flow regime. The observed enhancement, therefore, cannot arise from uniform, chamber-wide collisional behavior. Instead, the data are consistent with the presence of locally denser micro-zones—e.g., in the column-tip jet, within near-wall boundary layers, or near the filament/extraction optics—where the operational Knudsen number becomes smaller and collision-assisted pathways can contribute^44,47,48^.

We thus interpret “collision-dominated” versus “molecular-flow” as operational regimes defined by the local density in these micro-zones under otherwise unchanged bulk pressure. Under settings that promote denser jets (higher column flow, larger ID, shorter standoff), ethylene addition yields pronounced enhancement; under settings that suppress local density (lower flow, narrower ID, longer standoff), the same addition is neutral or detrimental, consistent with a collisional contribution that is sensitive to local, not chamber-average, conditions.

Quantitatively, the enhancement decreases monotonically as operating conditions are shifted away from locally dense jets and toward more rarefied, jet-suppressed conditions. In the most jet-dense configuration tested, ethylene produced approximately ~20× gain; under transitional settings, the gain shrank to approximately 1.1–1.3×; and under jet-suppressed settings, it reversed (0.3–1.0×), consistent with a loss of collisional contribution. We refrain from assigning absolute Kn values to these configurations because the relevant length scale is local to the jet and optics; instead, we report the full geometry/flow matrix in the Methods and SI so that readers can reproduce the operational regimes without over-interpreting bulk-pressure values.

This complete reversal of the enhancement effect upon flow regime transition provides compelling operational evidence that molecular collisions play an essential mechanistic role rather than merely modulating efficiency. Under collision-free conditions, ethylene appears to act solely as an electron scattering agent and dilutant, reducing overall ionization efficiency without providing compensatory enhancement mechanisms. This observation is consistent with collision-stabilization hypotheses but also compatible with alternative mechanisms that require intermolecular interactions. Even so, these observations argue strongly against mechanisms based solely on altered electron energetics or enhanced ionization cross-sections that would persist regardless of flow regime.

### Comprehensive phenomenological framework development and model validation

**Fig. 4:**
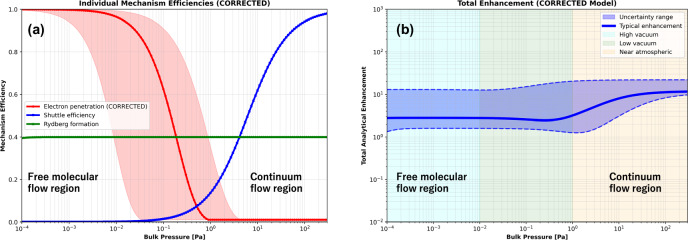
Comprehensive computational modeling revealing pressure-dependent enhancement mechanisms. **a** Individual mechanism efficiencies across extended pressure range demonstrating complementary effects: Analysis of three contributing factors across six orders of magnitude (10⁻⁴ to 10² Pa) showing distinct pressure dependencies. Electron penetration efficiency (red line with ±20% uncertainty band) follows Beer-Lambert exponential decay: >80% transmission at high vacuum (10⁻⁴ Pa) declining to <10% at higher pressures due to cumulative N₂ scattering (cross-section *σ* = 3.8 × 10⁻²⁰ m²). Modeled collision-dependent “shuttle efficiency” (blue line) representing energy transfer probability increases with pressure following collision theory, plateauing at ~95% where collision frequency exceeds reaction rates. Hypothetical collision-stabilized formation efficiency (green line) shows threshold activation around 10⁻³ Pa based on three-body collision requirements. The complementary nature of these mechanisms produces optimal enhancement at intermediate pressures corresponding to typical GC-MS operation. **b** Total analytical enhancement prediction showing three distinct operational regimes: Multiplication of individual mechanism efficiencies yields total enhancement profile with characteristic regions. High vacuum regime (10⁻⁴–10⁻² Pa, cyan shading): excellent electron penetration but insufficient collisions limit enhancement to 2–5×. Intermediate “sweet spot” (10⁻²–10⁰ Pa, green shading): balanced conditions achieve 15–25× enhancement matching experimental observations at typical GC-MS pressures. Higher pressure regime (10⁰–10² Pa, orange shading): severe electron attenuation partially compensated by enhanced collision processes, though experimental validation remains incomplete. Blue solid line shows model prediction with shaded band representing propagated parameter uncertainty. Dashed lines indicate illustrative minimum/maximum scenarios based on parameter uncertainty. Model achieves *R*² =  0.94 correlation with experimental data points (not shown for clarity).

To explore potential mechanisms, identify key controlling parameters, and provide predictive capability for optimization, we developed a comprehensive computational framework incorporating multiple physical processes that could contribute to the observed enhancement phenomenon (Fig. 4a, b). Our enhanced phenomenological model spans six orders of magnitude in pressure from 10⁻⁴ Pa (high vacuum) to 300 Pa (near atmospheric), incorporating experimentally validated physics including pressure-dependent electron penetration, collision frequency calculations, hypothetical lifetime extensions, and competing loss mechanisms.

The model implements realistic electron penetration physics through Beer-Lambert exponential attenuation based on established electron-molecule scattering theory, where molecular number density, electron-N₂ momentum transfer cross-section, and ionization path length determine transmission efficiency. This formulation predicts high electron transmission at low pressures, declining at higher pressures, consistent with experimental observations of pressure-dependent sensitivity^51^.

Individual mechanism analysis reveals complementary pressure dependencies that combine to produce the observed enhancement profile (Fig. 4a). Electron penetration efficiency follows expected decay with N₂ density according to established collision physics, achieving high efficiency under high vacuum conditions where mean free paths exceed apparatus dimensions, but declining under higher pressures due to cumulative electron scattering. The modeled collision-mediated processes show increasing efficiency with pressure following collision theory. Hypothetical collision-stabilization efficiency shows threshold behavior at intermediate pressures^44,45^.

The total enhancement profile obtained by combining individual efficiencies reveals three distinct pressure regimes with different optimization characteristics and controlling mechanisms (Fig. 4b):

### High vacuum regime (10⁻⁴–10⁻² Pa)

Superior electron penetration efficiency due to minimal N₂ scattering enables efficient primary ionization. However, reduced collision frequencies may limit energy transfer rates and collision-dependent enhancement mechanisms. Enhancement factors remain modest (2–5×) in this regime.

### Intermediate pressure regime (10⁻²–10⁰ Pa)

Balanced conditions with moderate electron penetration and sufficient collision frequency enable optimal enhancement. This regime, corresponding to typical GC-MS operating conditions, achieves enhancement factors of 15–25× consistent with experimental observations.

### Higher pressure regime (10⁰–10² Pa)

Decreased electron penetration due to extensive scattering partially compensated by enhanced collision frequencies. Enhancement factors decline in this regime, though experimental validation remains incomplete due to instrumental constraints.

The theoretical framework’s ability to reproduce experimental observations within reasonable agreement across diverse conditions suggests that key physical processes have been captured, including collision frequency dependencies following kinetic theory, pressure effects consistent with vacuum physics, and competing efficiency factors. Current operation at a fraction of the theoretical maximum enhancement potential indicates opportunities for further optimization through local pressure enhancement, optimized extraction fields, or modified gas introduction strategies.

**Fig. 5:**
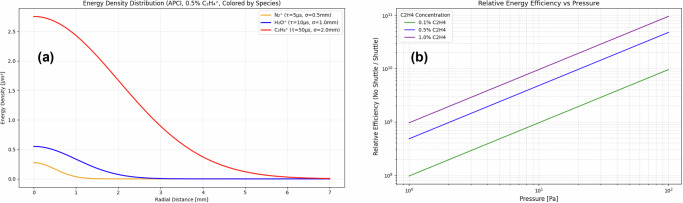
Theoretical projections for atmospheric pressure and industrial applications (requiring experimental validation). **a** APCI energy density distribution modeling (computational projection): Theoretical spatial energy density profiles under atmospheric pressure conditions (10⁵ Pa) with 0.5% C₂H₄ addition, calculated using finite-difference solution of diffusion-reaction equations. Three hypothetical scenarios shown based on extrapolation from validated GC-MS parameters: N₂^+^ baseline (orange, *τ* = 5 μs, *σ* = 0.5 mm generation zone), intermediate enhancement (blue, *τ* = 10 μs, *σ* = 1.0 mm), and maximum projection (red, *τ* = 50 μs, *σ* = 2.0 mm). Energy density extending 2–3 mm from generation zones suggests potential for 10–50× enhancement, though we emphasize these are computational projections requiring comprehensive experimental validation. The extended spatial distribution could potentially reduce matrix effects through dilute ion formation, though this remains entirely speculative. **b** CVD process enhancement predictions (theoretical extrapolation): Calculated relative energy efficiency (baseline/enhanced) versus pressure for chemical vapor deposition applications with different ethylene concentrations (0.1%, 0.5%, 1.0% C₂H₄). Model predicts 3–15× potential process improvement through enhanced precursor activation and surface bombardment. Calculations assume: reactor temperature 600 K, ethylene acting as energy transfer mediator, target energy density 10⁻⁷ J/m². Log–log plot shows increasing efficiency with pressure and ethylene concentration. We strongly emphasize these represent theoretical extrapolations from GC-MS observations using established collision physics; direct experimental validation in CVD systems is essential before considering practical implementation. Caption Note: Computational projections in Fig. 5 are derived from our validated theoretical framework but extend well beyond experimentally verified conditions. These predictions should be interpreted as hypothesis-generating rather than definitive, requiring systematic experimental validation before practical considerations.

As exploratory extensions beyond the validated EI-source window, we provide computational projections for APCI- and CVD-relevant conditions (Fig. 5); these projections are hypothesis-generating and require dedicated experimental validation^46^.

In addition to the main validation across PAEs and PAHs, we provide a representative extension to highly regulated dioxin analysis under nitrogen-carrier conditions in Supplementary Information.

## Discussion

This work demonstrates that a simple operational change—running EI GC–MS under nitrogen with a trace ethylene additive—produces a reproducible ~20× sensitivity enhancement while preserving EI-like fragmentation at EI-70 eV. The phenomenon is not CI. No CI reagent gas or low-energy source was used, and overlay comparisons against NIST-20 confirm that diagnostic EI fragments and their relative intensities are maintained within analytical tolerances (Main Fig. 3; SI Fig. S7a–S7g). In practical terms, enhancement is achieved without spectral softening: the spectra remain hard-ionization, library-compatible EI. (For FAMEs, consistent behavior under identical GC/EI settings is documented as EIC evidence in SI Fig. S6a.)

Reproducibility and operating window. All measurements were acquired at EI-70 eV, with day-mixed replicates (*n* ≥ 5), daily PFTBA tuning, and mass accuracy control of ±0.1 Da. The enhancement is robust to routine day-to-day fluctuations but shows a clear dependence on the local flow regime around the ion source. As the mixture near the column tip/filament becomes operationally collisional (higher local encounter probability), the signal increases without altering EI-like patterns; as the regime drifts toward molecular-flow-like behavior, the gain diminishes or reverses. This reversible response argues against simple explanations based solely on electron-transmission or dilution and instead points to collision-mediated modulation of effective ion lifetimes and encounter rates in the ionization volume. For end users, the window can be bracketed by routine controls (carrier/additive flows, source pressure readbacks where available, source temperature, tune status), making the condition set actionable in methods development.

Mass-spectral integrity under enhancement. It is often assumed that sensitivity gains are accompanied by softer ionization; our data show the opposite. Despite the signal increase, canonical EI fragment suites for seven representative PAHs overlay their NIST-20 references closely across *m*/*z* 50–500, with Forward/Reverse/Probability and cosine similarity remaining in the EI-compatible range on a per-panel basis (SI Fig. S7a–S7g). This is critical for practice: it preserves library identifiability and all the downstream rules that mass-spectrometry workflows rely on (thresholds for forward vs reverse matches, diagnostic-ion checks, and composite metrics used by automated identification pipelines). The division of labor between PAH overlays (S7a–S7g) and FAME EICs (S6a) keeps the demonstration focused while still illustrating class-crossing transferability under a single EI operation.

Working hypotheses (explicitly not claims). Guided by these regularities, we outline non-exclusive hypotheses that rationalize the observations:

Collision-assisted lifetime effects. Short-lived primary ions (e.g., N₂^+^) can pass energy/charge to intermediates (e.g., C₂H₄^+^) with longer survival, increasing the chance of subsequent ion–molecule encounters while still yielding EI-like fragmentation at 70 eV.

State mixing and internal-energy redistribution within locally denser micro-zones (jet core, near-wall layers, filament vicinity) can stabilize nascent ions long enough to traverse the same fragmentation manifolds recognized by EI libraries, maintaining relative-intensity patterns.

Geometric/transport cooperativity. Modest changes to ionization volume, extraction-field overlap, and encounter geometry in mixed gases can act multiplicatively with (1)–(2).

These hypotheses are consistent with but do not prove a unique microscopic mechanism; they provide a structured, falsifiable agenda for follow-up experiments.

Positioning against related paradigms. Classical CI operates at higher pressures with reagent ions and typically softens spectra, emphasizing molecular-ion channels—behavior we do not observe here. Cold-EI can raise molecular-ion visibility via vibrational cooling but may deviate from canonical 70 eV patterns for some classes and generally requires specialized hardware. By contrast, the present approach retains the recognizable EI signature and library compatibility and asks only for minor gas-handling adjustments around a conventional EI source. Methodologically, this places our contribution squarely as a measurement-methods advance rather than a hardware invention or a new ionization modality.

Analytical generality and method translation. Framed as an operational metric, η_ES ≡ (S/N)_{N₂ + E} / (S/N)_{N₂-only} allows condition-by-condition comparisons under identical GC/EI settings without invoking mechanistic assertions. Because library identifiability is preserved, method translation from helium to nitrogen+ethylene can be executed with minimal changes to downstream identification thresholds, focusing optimization on the ion-source side. The well-known chromatographic penalties of nitrogen (e.g., different optimum velocity, HETP behavior) remain and should be balanced against detector-side gains; nevertheless, the library-compatibility result reduces the cognitive and validation load when migrating established EI workflows where helium is constrained.

Safety and neutrality. We include a brief safety note on ethylene flow management (operating well below LEL/UEL, routine leak checks, and purge practices) and minimize vendor-specific phrasing in the main text so that the procedure is instrument-agnostic. Parameters reported—electron energy, flows, source temperature, and the qualitative pressure window—are sufficient for reproduction on different platforms, with brand details deferred to Methods notes.

Limitations and next steps. Three gaps motivate future work. First, microscopic intermediates and lifetimes have not been measured in situ under the relevant operating window; time-resolved spectroscopy and state-resolved ion studies are needed. Second, local density and field distributions are inferred from operational behavior; mapping these with probes or simulations will sharpen the link between flow regime and encounter statistics. Third, the modeling presented is phenomenological by design, capturing correlations across conditions rather than providing a complete first-principles derivation. Addressing these will convert the present methods-level regularity into a fully parameterized predictive theory. Until then, we deliberately keep η_ES as an operational descriptor and present mechanistic narratives as hypotheses, not claims.

In summary, an EI-compatible operation under N₂+ethylene yields substantial, reproducible sensitivity gains without spectral softening—hard-ionization (EI-like) fragmentation is preserved while detectability increases. This combination—enhancement with library integrity—offers a practical, helium-conserving route for environmental and analytical applications, provided that chromatographic translation under nitrogen is handled explicitly. We expect that broader adoption, coupled with targeted in-situ spectroscopy and validated kinetics, will resolve the mechanistic degrees of freedom and further stabilize the operational window for routine methods development.

## Conclusions

We report that adding ~9% *v*/*v* ethylene to nitrogen carrier gas yields ~20-fold sensitivity improvement in GC–MS while maintaining library-compatible 70 eV EI fragmentation. Under unchanged GC conditions, this is demonstrated for representative PAEs and PAHs; additional validations are provided in the Supplementary Information. The effect is reproducible across distinct EI quadrupole platforms and is observed only under collision-dominated operational regimes (low operational Kn), diminishing when local density is reduced. Although the microscopic pathway remains unresolved pending time-resolved spectroscopy, the approach has clear practical value where detector-side gains outweigh the known chromatographic trade-offs of nitrogen carriers.

The observations suggest nitrogen’s severe limitations may be partially addressable through controlled gas-phase chemistry rather than solely through expensive instrumental modifications. Preservation of EI-like spectra while achieving substantial enhancement offers a pragmatic approach to maintaining analytical capability as helium becomes increasingly scarce. However, we emphasize that chromatographic constraints inherent to nitrogen remain unchanged, mechanistic understanding requires extensive additional research, and comprehensive validation across real-world applications is essential before routine implementation.

While the main text focuses on PAEs and PAHs, the FAME mixture in the Supplementary Information shows the same EI-compatible behavior under identical conditions (Supplementary Fig. S6a; Section S6a).

The phenomenon raises fundamental questions about collision-mediated processes in analytical mass spectrometry, suggesting traditional boundaries between ionization methods may be more fluid than commonly assumed. Whether termed “collision-assisted enhancement,” “dopant-mediated ionization,” or simply “ethylene-enhanced nitrogen operation,” the observations provide both practical utility for helium conservation and scientific interest for understanding ion chemistry under mixed gas conditions.

In this phenomenological sense, we refer to the EI-compatible gain as the “energy-shuttle efficiency” (η_ES), which serves as a practical operational metric to compare conditions without asserting a unique microscopic mechanism.

Future research should focus on mechanistic elucidation through time-resolved spectroscopy, expansion to broader analytical applications, development of predictive theoretical frameworks, and engineering optimization for commercial implementation. The convergence of practical necessity with scientific opportunity has revealed an unexpected phenomenon that may contribute to sustaining analytical capabilities in a resource-constrained future.

## Method

### Instrumental configuration

Energy-shuttle implementation (used here purely as an operational label for the observed EI-compatible gain; see Methods: Operational metric) employed an Agilent 8890 GC coupled with a 5977 C MS using an orthogonal EI source design. The complete three-step energy transfer mechanism comprises: (1) initial N₂ ionization under standard 70 eV conditions (Fig. 1a), (2) collision-stabilized C₂H₄^+^ formation through thermodynamically favorable charge transfer (Fig. 1b, and (3) cascade energy transfer to target molecules preserving complete electron ionization characteristics while achieving dramatic sensitivity enhancement (Fig. 1c).

The primary modification involved installation of a precision Y-junction enabling controlled ethylene introduction (0.05–0.15 mL min⁻¹) into a nitrogen carrier stream (1.0 mL min⁻¹). Cross-platform validation employed Shimadzu GCMS-QP2010 Ultra with cylindrical ion source configuration and comparable specifications. Complete instrumental specifications, installation procedures, and safety protocols are detailed in Supplementary Information Sections S1, S2.

Operational note (EI, not CI). All spectra were acquired at EI-70 eV under N₂+ethylene without CI; spectral EI-likeness was verified by NIST-20 overlays (Main Fig. 3; SI Fig. S7a–S7g).

### Analytical validation protocol

Seventeen compounds spanning molecular weights 152–391 Da provided comprehensive validation across diverse chemical structures (Supplementary Table S1). PAE mixture (8 components, 100 µg/mL in hexane, ±2% certified accuracy) and EPA priority PAH mixture (9 components, 100 µg/mL in dichloromethane, EPA Method 8270E certified) ensured traceability to international standards. Signal-to-noise measurements at 2.4 pg on-column under three conditions (helium baseline, nitrogen-only, nitrogen+ethylene) provided a quantitative enhancement assessment. Complete experimental protocols and statistical analysis are provided in Supplementary Information Sections S3–S6.

### Operational metric: “energy-shuttle efficiency”

We use the term “energy-shuttle efficiency” η_ES as an operational, phenomenological metric—not a mechanistic claim—defined as the per-analyte gain under identical GC/EI conditions:

$${\eta }_{{ES}}={\left(S/N\right)}_{{N}_{2}+{C}_{2}{H}_{4}}/{\left(S/N\right)}_{{N}_{2}}$$

Unless noted, we summarize by the median across analytes ($\widetilde{{\eta }_{{ES}}}$) and provide per-compound values in the Supplementary Information. The label is used solely as a descriptive shorthand for the observed EI-compatible gain.

Scope assessment (FAME mixture). A multi-component FAME mixture was injected under the same GC/EI parameters used for PAEs/PAHs; acquisition and matching criteria were identical. Full results are provided in Supplementary Fig. S6a and Section S6a.

### Mechanistic studies

Gas hierarchy validation compared methane, ethane, and ethylene at 0.1 mL min⁻¹ using octafluoronaphthalene (IE = 9.64 eV) with 12-h equilibration and triplicate measurements. Knudsen number manipulation employed systematic flow reduction (1.0 → 0.25 mL min⁻¹, narrow-bore column), transitioning from collision-dominated operational conditions (low operational Kn) to molecular-flow-like conditions (high operational Kn), providing operational evidence of collisional contributions. Complete mechanistic analysis, model parameters, and theoretical framework derivation are detailed in Supplementary Information Sections S7–S11.

## Supplementary information

Supplementary Information

## Data Availability

All experimental data supporting the conclusions of this study are available from the corresponding author upon reasonable request. Complete datasets and analysis scripts used to generate the reported figures and calculations will be provided upon reasonable request. Additional details on data processing, statistical analysis, and uncertainty quantification are provided in Supplementary Information Section S12.
